# An overview of literature on COVID-19, MERS and SARS: Using text mining and latent Dirichlet allocation

**DOI:** 10.1177/0165551520954674

**Published:** 2022-06

**Authors:** Xian Cheng, Qiang Cao, Stephen Shaoyi Liao

**Affiliations:** Business School, Sichuan University, China; Department of Information Systems, City University of Hong Kong, China; Department of Information Systems, City University of Hong Kong, China

**Keywords:** COVID-19, latent Dirichlet allocation, literature analysis, MERS, SARS, text mining

## Abstract

The unprecedented outbreak of COVID-19 is one of the most serious global threats to public health in this century. During this crisis, specialists in information science could play key roles to support the efforts of scientists in the health and medical community for combatting COVID-19. In this article, we demonstrate that information specialists can support health and medical community by applying text mining technique with latent Dirichlet allocation procedure to perform an overview of a mass of coronavirus literature. This overview presents the generic research themes of the coronavirus diseases: COVID-19, MERS and SARS, reveals the representative literature per main research theme and displays a network visualisation to explore the overlapping, similarity and difference among these themes. The overview can help the health and medical communities to extract useful information and interrelationships from coronavirus-related studies.

## 1. Introduction

**Figure 1.**
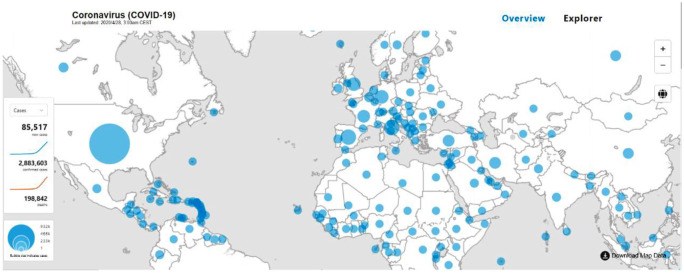
The geographical distribution of confirmed cases for COVID-19. Last updated: 28 April 2020 [2].

The unprecedented outbreak of coronavirus disease 2019 (COVID-19) [1], caused by a novel coronavirus named severe acute respiratory syndrome coronavirus 2 (SARS-CoV-2), represents one of the most substantial global challenges in this century. The pandemic has severe consequences for public health, economics, politics and society. On 28 April 2020, about 180 countries and territories reported a combined total of about 2,883,603 laboratory-confirmed cases, with 198,842 deaths globally [2]. Figure 1 presents the geographical distribution of COVID-19 confirmed cases. SARS-CoV-2, taxonomically, is currently classed as a species of SARS-related coronavirus and belongs to the genus *Betacoronavirus* [3]. Two others similar betacoronaviruses, SARS-CoV and MERS-CoV, have also caused epidemics around the world in the last two decades, specifically SARS in 2002–2003 and the Middle East respiratory syndrome (MERS) in 2012–2013. Several similarities and differences in the causative agents, pathogenesis and immune responses, epidemiology, diagnosis, treatment and management of COVID-19, SARS and MERS have been identified [4–6]. For example, Law et al. [6] discuss the current understanding of COVID-19 and compare it with the outbreak of SARS in 2003 in Hong Kong in terms of the causes, transmission, symptoms, diagnosis, treatments and preventions, to establish an effective measure to control COVID-19.

In response to the COVID-19 pandemic, a large number of academic studies and case reports have already emerged in major international scientific and medical journals. Most of them addressed relevant research questions, including the virus’s evolution and effects, as well as potential risk factors and clinical, laboratory and imaging findings [7]. In addition, to support the efforts of scientists in the health and medical community in combatting COVID-19, many leading research organisations created a range of free resources for scholars and the public to download and read. For example, in support of the global efforts in diagnosis, treatment, prevention and further research on SARS-CoV-2 and COVID-19, Elsevier has established the Novel Coronavirus Information Center and made more than 24,000 related articles free to access on ScienceDirect [8]. Another example is Kaggle, which has launched the COVID-19 Open Research Dataset (CORD-19), containing over 57,000 scholarly articles, including over 45,000 with full text, on COVID-19, SARS-CoV-2 and related coronaviruses [9]. However, the huge amount of coronavirus literature from numerous information sources can be difficult for the health and medical community to keep up with. It is vital to establish how a literature review on these coronavirus studies can be performed most rapidly, and how the main research themes for COVID-19 can be classified. As the COVID-19 research efforts build on earlier research on SARS and MERS, one can expect both similarities and differences among the research themes related to COVID-19, MERS and SARS. Although it is vital for the health and medical community to understand coronavirus-related diseases, answering research questions will be very challenging. First, it is impossible to categorise the vast quantity of disparate literature from this rapidly growing subject area through manual processes, as the time frame involved increases linearly with the volume of literature under analysis [10]. Besides, manual categorisation of the coronavirus literature into major research themes could be prone to various biases. However, with the rise of information and communication technology (ICT) in information science, the widespread recent developments in data mining technologies, particularly text mining techniques, offer potential solutions to these challenges by allowing analysis of a large number of unstructured documents through automated processes [11]. Indeed, the vast amount of coronavirus literature provides the ideal arena for specialists in information science to apply text mining techniques to find relevant answers to research questions and synergise existing research insights for the health and medical community [12].

Text mining, which comprises a range of techniques such as latent Dirichlet allocation (LDA), together with natural language processing, can be used to identify and extract information or relationships from unstructured data and has become a popular approach to literature analysis in an era of rapidly emerging research [13–15]. For example, Ozaydin et al. [11] performed a comprehensive literature review of mobile health services from 5644 research articles using text mining. LDA, which is a Bayesian probabilistic model of text documents according to ‘bag of words’ [16] and generates the proper topics from documents by utilising a probability distribution to ensure all topics obey a Dirichlet polynomial prior distribution [17], is widely used in literature analysis. In this article, we combine the application of text mining with LDA procedure to perform a literature analysis of the coronavirus literature and provide an overview of the research that has been conducted on COVID-19 and other coronavirus-related pneumonias (MERS and SARS). In detail, the main purposes of this article are as follows:

- To identify the most relevant search terms and generic research themes of three coronavirus diseases – COVID-19, MERS and SARS – by performing an automated literature analysis and synthesis based on text mining and LDA.
- To uncover the representative literature on each main research theme for coronavirus-related diseases, thereby helping the health and medical community to find the appropriate studies on target themes for these diseases.
- To build a novel visual concept network that visualises the similarities among the research themes for coronavirus diseases to reveal the key aspects of these pathogens and the extent of overlapping, similarity and difference among these themes.

The first contribution of the study is to present an overview of coronavirus literature using text mining for coronavirus-related research, offer a structured morphology of the existing literature, uncover the research themes and representative literature for each theme, and reveal the overlapping, similarity and difference among these themes. Our literature analysis can help the health and medical communities to combat COVID-19 by facilitating the extraction of useful information and interrelationships from the mass of coronavirus literature. The second contribution is to propose a methodological framework for science foresight analysis [18]. The framework rapidly provides a snapshot of any specific field of study, enabling scholars to evaluate possible opportunities for new research and development activities in their field.

This article is organised as follows. In section 2, we introduce the main concepts of infectious diseases caused by coronaviruses and the related research in the form of literature analysis and synthesis and present some literature on text mining. In section 3, we present the data and methods used in this research. In section 4, the results are analysed and discussed. Finally, in section 5, we summarise our conclusions and present future research directions.

## 2. Background

### 2.1. Coronaviruses and related diseases

Belonging to the *Coronaviridae* family, coronaviruses are a group of enveloped, single-stranded RNA viruses present in various species of birds, snakes, bats and other mammals. According to their serological pattern, coronaviruses can be grouped as alpha, beta, gamma and delta [19]. Diseases caused by coronavirus infection have emerged as epidemic and pandemic outbreaks more than once in the last few decades. Outbreaks in humans have been caused by infection with various coronaviruses, including 229E, OC43, NL63, HKU1, SARS-CoV and MERS-CoV. The recent SARS-CoV-2 has proved to be the most serious coronavirus to date, as it has spread across 203 countries and territories in all five major continents. All coronavirus diseases produce similar symptoms such as rhinorrhea, mild or severe cough, tracheitis and bronchitis [6]. SARS-CoV, MERS-CoV and the recently discovered SARS-CoV-2 are all grouped as betacoronaviruses.

SARS-CoV was identified in 2003. From November 2002 to March 2003, about 8096 people were affected in 26 countries. With 774 deaths by the end of May 2004, the mortality rate was approximately 9.56% [20]. Genetic analysis shows that SARS-CoV has a nucleotide sequence similarity to other coronaviruses of only about 50%–60%. SARS-CoV also has a high mutation rate, and can still be cultured after residing on various surfaces for up to 24 h [21]. Bats have been found to harbour SARS-CoV and transmit it to human hosts [22]. However, the transmissibility of SARS-CoV is lower than that of SARS-CoV-2.

MERS-CoV, which originated from camels [23], was first discovered in the Middle East countries (Saudi Arabia, Oman, UAE) in 2012 when a cluster of cases of respiratory tract infection started to surface. MERS-CoV subsequently spread to 24 other countries, including Malaysia and the United States, and genetic analysis revealed some homology with SARS-CoV [24]. From September 2012 to 30 June 2018, about 2239 confirmed cases of MERS-CoV were reported by the World Health Organization (WHO). About 83% of the cases came from Saudi Arabia, and the crude fatality rate was 35.5% during this period, including 791 individuals who died due to other co-morbid illnesses, such as diabetes, renal failure and hypertension [25].

### 2.2. Literature analysis

Involving searching, screening and synthesising research materials from multiple sources, the literature analysis is a structured methodology to evaluate a body of literature to inform research development, identify potential research gaps and highlight the boundaries of a research subject [26]. Literature analysis enhances the effectiveness of the management and planning of research and development activities [18]. The typical process flow of a literature analysis involves defining appropriate search keywords, searching the literature and completing the analysis [27]. Traditionally, literature analysis required considerable efforts from domain experts. Although online library databases enable researchers easily to search an enormous amount of available articles from any physical location, the high volumes of articles returned presently the challenging task of reading and analysing the contents of each paper, even though only a small part of some articles may be relevant [28]. Today, new technologies such as text mining are used in literature analysis.

In biomedicine, new research heavily depends on making full use of previous scientific work, so literature analysis is a crucial tool for biomedicine. Table 1 presents a summary of several selected works from a literature analysis of biomedical articles. Four of the articles concern coronavirus-related infectious diseases: two for COVID-19 and two for SARS. The literature analytical techniques used include meta-analysis, qualitative or quantitative analysis and citation analysis. The last three articles focus on health gamification, telemedicine and cognitive computing in healthcare.

**Table 1.** Selected articles for biomedical literature analysis.

Reference	Research areas	Number of relevantliteratures	Search period	Techniques have been used
Rodriguez-Moraleset al. [3]	COVID-19	27	1 January 2020 to 23 February 2020	Meta-analysis, qualitative and quantitative analysis
Harapan et al. [29]	COVID-19	70	–	Summary method
Kostoff [30]	SARS	2874	2003–2008	Citation analysis, literature survey, Citation-Assisted Background (CAB)
Kostoff and Morse [31]	SARS	2874	2002–2008	Text mining, bibliometrics, citationanalysis
Alahäivälä et al. [32]	Health gamification	15	2012–2015	Persuasion context analysis
Armfield et al. [33]	Telemedicine	17,932	1970–2013	Bibliometric analysis, content analysis
Behera et al. [34]	Cognitive computingin healthcare	7700	2014-2018	Content analysis, WordCloud

### 2.3. Text mining

As a particular type of data mining, text mining aims to extract useful knowledge such as relations, patterns and trends from unstructured or semi-structured data, for example, text documents [35,36]. The main process in text mining is transforming text into numerical data using statistical methods to extract textual contents into an organised document-term matrix, which encompasses the following two dimensions: the words (or terms, composed of *n* words) and the documents [37]. The two most common techniques developed in recent years for building knowledge using text mining are latent semantic analysis (LSA) and topic modelling. LSA is a form of natural language processing that extracts relationships between textual terms and documents by assuming that words with similar meaning will occur in similar pieces of text [38]. Topic modelling transforms the relevant words and their frequency into an organised structure, in which the documents are distributed into several topics [39]. There are many variants of those techniques: for example, the work of Lee et al. [40] presents a comparative study of four techniques in text mining, including two LSA techniques (LSA and probabilistic latent semantic analysis (PLSA)) and two topic modelling techniques (LDA and correlated topic modelling). The authors highlight that LDA is the best tool for dealing with multiple topics. This technique can determine the probability of each document belonging to each of the topics, and groups the documents into the most probably matching topics [41].

Text mining is now widely applied in biomedical research, as a vast number of biomedical texts, such as electronic patient-authored texts [42] and biomedical studies [43], provide a rich source of knowledge. Text mining effectively empowers researchers to create new information by making use of existing biomedical work. In biomedical literature analysis, there is a pressing need to deploy new technology that can automatically extract knowledge from published literature in response to the recent double exponential growth rate of biomedical literature [44]. Text mining is a suitable technique for such a challenge. Table 2 presents several selected papers on text mining–based approaches for biomedical study.

**Table 2.** Text mining–based approach for biomedical study.

Reference	Research areas	Technique	Data type	Objective	Literature analysis
Hashimoto et al. [45]	Biomedicalsystematicreview	Topic modelling, active learning	Clinical public healthreviews	To detect topics	No
Kim and Delen [43]	Medicalinformatics	Content analysis, cluster analysis	Medical informaticsliterature	To investigate major subject areas	Yes
Ozaydin et al. [11]	Mobile healthresearch	Natural languageprocessing, clusteranalysis, WordCloud	mHealth literature	To analyse the evolution of mHealth research	Yes
Singhal et al. [46]	Precisionmedicine	Automated extraction, machine learning	Biomedical literature repositories	To automatically identify disease-mutation relationships	No
Kostoff et al. [31]	SARS	Computationallinguistics,clustering analysis	SARS research literature	To identify preventive measures and treatments	Yes
Lucini et al. [47]	Emergency beddemands	Feature selection	Textual medicalrecords	To develop a prediction model of inpatient bed demand	No
de Bruijn andMartin [48]	Molecularbiology	Natural languageprocessing,collection-wideanalysis	Biomedicalliterature	To give developments in medical language processing	Yes

As a Bayesian probabilistic model for identifying latent topics from large and unstructured text documents, LDA is one of the most widely used topic modelling tools in literature analysis. For example, Wu et al. [17] employed LDA to perform topic segmentation and topic evolution for literature on stem cell research. By proposing a topic analysis approach incorporating LDA and the three-dimensional strategic diagram, Feng et al. [16] analysed the 62,340 literatures in the field of medical informatics between 1991 and 2018. By following LSA [38] and PLSA [49], LDA was first proposed by Blei et al. [41] in 2003 and adopted the Dirichlet prior distribution with the assumption that all topics are uncorrelated. LDA has several advantages for literature analysis. First, LDA is highly efficient for dealing with big data as it can capture effectively text-specific dimensions and does not make any assumption [50]. Second, LDA incorporates several steps of text analysis with little human intervention, for example, data sampling, and thus the result of topic modelling is more realistic and objective.

## 3. Data and methods

### 3.1. Data description

#### 3.1.1. Data sampling

We conduct text mining based on CORD-19, which contains more than 57,000 scholarly papers (43,540 full texts) about COVID-19, MERS, SARS and other coronavirus diseases [9]. This data set is updated regularly and includes peer-reviewed publications and preprint literature from PubMed Central, bioRxiv, medRxiv and others. The latest update date of the data set in this study is 24 April 2020.

To focus on the three studied coronavirus diseases, COVID-19, MERS and SARS, we search for studies with matched keywords in the titles as well as the abstracts. The keywords for COVID-19 are ‘COVID-19’, ‘SARS-CoV-2’, ‘2019-nCoV’, ‘novel coronavirus pneumonia’ and ‘novel coronavirus infected pneumonia’. The keywords for MERS are ‘MERS’ and ‘Middle East respiratory syndrome’, and those for SARS are ‘SARS’ and ‘severe acute respiratory syndrome’. After keyword matching, we exclude several irrelevant studies by manual inspection. Only English literatures are included. Finally, we have 3440 studies related to COVID-19, 1590 studies related to MERS and 2879 related to SARS, and the total number of literatures is 7909. These studies are published in 1461 journals. We list the top 20 journals by publication number in Table 3.

**Table 3.** Top 20 publication journals.

Journal name	Publication number
*Emerging Infectious Diseases journal*	228
*PLOS One*	136
*Antiviral Research*	116
*Virology*	115
*Biochemical and Biophysical Research Communications*	81
*Virus Research*	79
*Viruses*	79
*International Journal of Infectious Diseases*	78
*Vaccine*	68
*The Lancet*	62
*Clinical Infectious Diseases*	54
*Scientific Reports*	52
*BMC Infectious Diseases*	51
*Emerging Microbes & Infections*	51
*Virology Journal*	51
*mBio*	49
*PLOS Pathogens*	49
*Journal of Infection*	48
*The Lancet Infectious Diseases*	48
*Journal of Medical Virology*	47

#### 3.1.2. Publication trends

We summarise the publication trends of the literature on the three coronavirus diseases in the form of a publication number bar chart. The *x*-axis represents the publish time (those for COVID-19 are reported monthly, while the other two are reported by year). The *y*-axis represents the number of publications.

**Figure 2.**
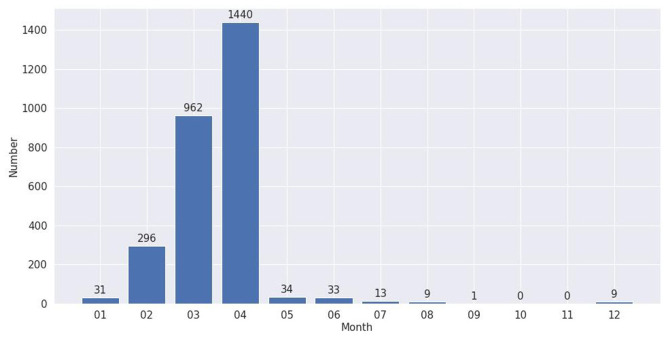
Number of COVID-19-related literatures, 2020.

The first case of COVID-19 was reported in Wuhan, China, in late December 2019 [47]. In our literature collections, the earliest academic study related to COVID-19 was published in January 2020. Because of the rapid growth of infected cases, the WHO declared a Public Health Emergency of International Concern on 30 January [2]. On 11 March, the WHO assessed COVID-19 as a pandemic [1]. A resulting boom in research literature after February 2020 can be identified in Figure 2. As the latest update time of the data set in this study is 24 April 2020, most of the literatures are published from January to April. Some of the studies even appeared in December 2020 issues of journals (December 2020 is the publish time, not the submit time).

**Figure 3.**
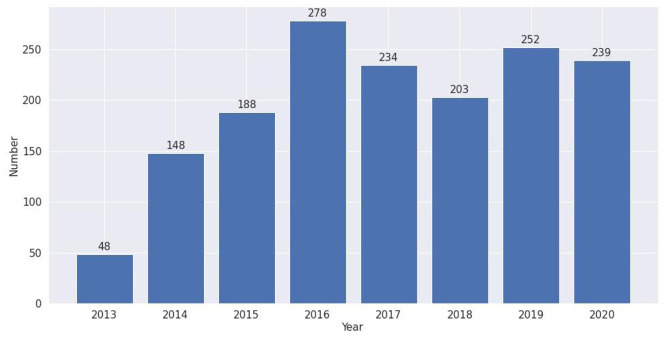
Number of MERS-related literatures, 2013–2020.

The first confirmed case of MERS occurred in 2012. Two later outbreaks occurred in South Korea in 2015 and Saudi Arabia in 2018 [23]. As research usually requires several months to 1 or 2 years to complete, we find two publication peaks in 2016 and 2019 in Figure 3. Based on the trend for the first quarter of 2020, we can expect another publication peak this year.

**Figure 4.**
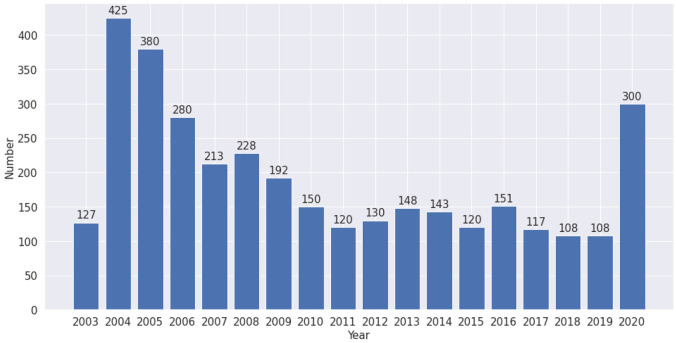
Number of SARS-related literatures, 2003–2020.

The outbreak of SARS was reported in 2003 [48]. In Figure 4, we find a peak in 2004, again because research and publication take some time. After 2004, the number of publications decreased until 2016, 1 year after the outbreak of MERS. We also find an increase in 2020 because of the outbreak of COVID-19.

### 3.2. Proposed methods

LDA is one of the most popular topic modelling methods [49]. Three concepts are important when applying the LDA algorithm: corpus, documents and terms. We refer to the total text collection as the corpus. Every item within the corpus can be considered as a document. Words in a document are called terms. Here, we consider documents as a mixture of latent topics. Latent topics can be inferred by modelling the distribution of words. Expressed another way, topics can be seen as items composed of a group of words. Documents are then composed of topics with different weights [50]. In detail, a literature is a document W which is a set of *n* words represented by $W=(\omega_{1},\omega_{2},\ldots ,\omega_{n})$, where $\omega_{n}$ is the *n*th word in the document; the set of *M* documents constitutes a corpus *D* which is denoted by $D=(W_{1},W_{2},\ldots ,W_{M})$. LDA assumes that the corpus *D* contains *K* topics, and each topic defines a multinomial distribution. Based on Blei et al. [41], the process for LDA is presented as follows:

First, the Dirichlet distribution η and θ in the selection process are defined: θ with parameter α for word selection and η with parameter β for topic section.
Second, the general process for each document W is described in the following two steps:

1. Choose θ~Dir(β).
2. For each of the *n* works $\omega_{n}$:
  - Choose a topic $z_{n}\sim \mathrm{Multinomial}(\theta )$.
  - Choose a word $\omega_{n}$ from $p(\omega_{n}|z_{n},\beta )$, a multinomial probability conditioned on the topic $z_{n}$.

**Figure 5.**
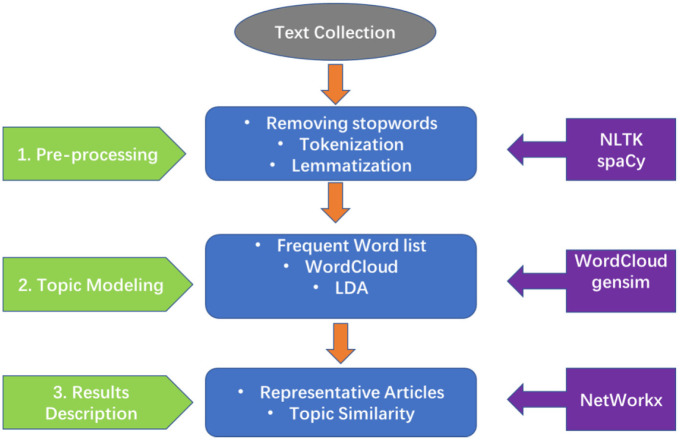
The proposed approach for text mining.

In this research, we use body text of literatures to conduct the experiments. The proposed text mining methods are displayed in Figure 5. Before conducting LDA, some pre-processing tasks are required. We use two Python libraries – natural language toolkit (NLTK) and spaCy (Industrial-Strength Natural Language Processing in Python) – for the data pre-processing. Data pre-processing includes the following three steps: (1) removing punctuation, unnecessary special characters and stop words; (2) tokenisation, that is, chopping the documents up into words; and (3) lemmatisation, that is, removing inflectional endings to retrieve the root or dictionary form of a word. After removing the other forms of words, only nouns and adjectives are left. We also include bigram [50] words in the data to extract more valuable information. A bigram is a set of two adjacent words: for example, ‘machine’ and ‘learning’ could be combined into the bigram ‘machine_learning’.

After pre-processing, we present the top 30 most frequent words for each of the three diseases. WordCloud, a popular Python visualisation tool, is also used to display the frequency of terms in the three disease-related literature corpora. We then use the LDA module in Gensim, a widely used topic modelling library, to extract meaningful topics from the collection of documents [35]. We also display the most relevant publications in each topic as well as the top three most frequent terms for the topic. Finally, we calculate the semantic similarity among different topics. We use NetworkX, a popular network visualisation tool, to display the semantic similarity network.

## 4. Results and analysis

The presentation of results is divided into three sections: topic modelling, representative studies and topic similarity networks. In section 4.1, we present the top 30 most frequent words associated with each of the three diseases. WordCloud is also used to display the most frequent terms in the literature corpora related to the diseases. We then present the topic modelling results. In section 4.2, we identify the most relevant literature for each topic and the topics’ top three most frequent terms. Finally, we calculate the semantic similarity among the topics in section 4.3.

### 4.1. Topic modelling results

#### 4.1.1. Most relevant terms

**Figure 6.**
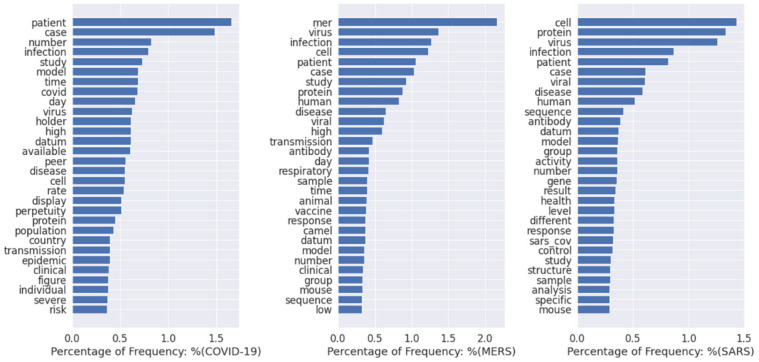
The most relevant terms for coronavirus-related diseases. ‘covid’ refers to the COVID-19; ‘mers’ refers to the MERS; ‘sars_cov’ refers to the SARS-CoV.

First, we present the global results for the text mining of coronavirus-related disease literature. Table 4 shows the frequencies of the most relevant terms for the three coronavirus-related diseases (COVID-19, MERS and SARS). Here, we only present the top 30 most relevant terms due to limited space. From this table, we can discover that the most relevant terms for research on the three coronavirus-related diseases include ‘patient’, ‘case’ and ‘infection’. This indicates that there are some similar research directions for the three diseases, consistent with the fact that they are all caused by coronavirus infection. In addition to these research commonalities, there also exist research differences among the three diseases. Specifically, the top three terms for COVID-19 are ‘patient’, ‘case’ and ‘number’. This reveals that the current research on COVID-19 mainly focuses on the symptoms of patients or the number of infection cases. This indicates that medical specialists still have a far from sufficient understanding and knowledge of SARS-COV-2. This is again to be expected, as the outbreak of this disease is very recent. For MERS-related research, although ‘MERS’, ‘virus’ and ‘infection’ are the most frequent terms, the fourth term is ‘cell’, which reveals that MERS research also now heavily concentrates on the study at the cellular level, for example, the status of infected cells. ‘Camel’ is another frequent term in MERS-related research, presumably related to the region/countries in which outbreaks occur. For SARS, it is evident that infection cases are not the primary concern for current research, as the top two terms are ‘cell’ and ‘protein’, which indicate that protein and antibody–related research is more prevalent for SARS. We also present the percentage frequency of the most popular terms in Figure 6, which allows the visualisation of the results.

**Table 4.** Top 30 frequent terms for research on coronavirus-related diseases.

Number	COVID-19		MERS		SARS	
	Term	Frequency	Term	Frequency	Term	Frequency
1	patient	40,297	MERS	39,122	cell	59,433
2	case	36,107	virus	24,661	protein	55,537
3	number	19,918	infection	22,963	virus	52,250
4	infection	19,254	cell	22,187	infection	35,964
5	study	17,663	patient	19,079	patient	33,843
6	model	16,693	case	18,598	case	25,490
7	time	16,581	study	16,743	viral	25,195
8	COVID-19	16,425	protein	15,914	disease	24,233
9	day	15,907	human	15,000	human	21,486
10	virus	15,062	disease	11,768	sequence	17,104
11	holder	14,873	viral	11,339	antibody	15,975
12	high	14,811	high	10,890	datum	15,418
13	datum	14,808	transmission	8532	model	15,254
14	available	14,659	antibody	7594	group	14,978
15	peer	13,423	day	7561	activity	14,957
16	disease	13,380	respiratory	7528	number	14,872
17	cell	13,367	sample	7237	gene	14,796
18	rate	13,055	time	7149	result	14,340
19	display	12,419	animal	7004	health	13,880
20	perpetuity	12,390	vaccine	6872	level	13,870
21	protein	10,887	response	6807	different	13,634
22	population	10,457	camel	6770	response	13,575
23	country	9591	datum	6691	SARS-CoV	13,308
24	transmission	9573	model	6446	control	13,235
25	epidemic	9553	number	6439	study	12,431
26	clinical	9263	clinical	6085	structure	12,301
27	figure	9087	group	6023	sample	12,290
28	individual	9010	mouse	5989	analysis	12,094
29	severe	8974	sequence	5860	specific	12,069
30	risk	8817	low	5793	mouse	12,038

#### 4.1.2. WordCloud for coronavirus diseases

We use WordCloud to display the most important terms in the corpora on the three coronavirus-related diseases. The font size in the figure depends on the term frequency without lemmatisation or bigram processing. Therefore, the word frequency of WordCloud is slightly different to the LDA model.

**Figure 7.**
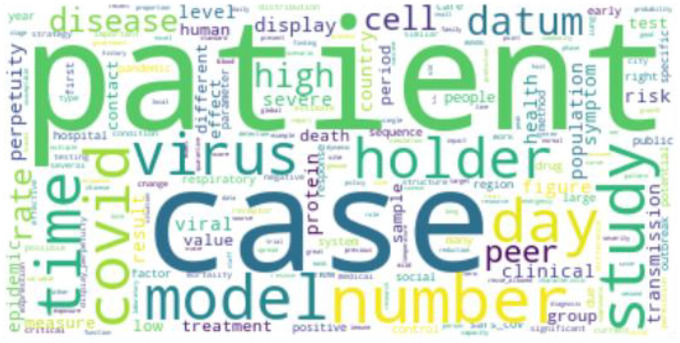
WordCloud of COVID-19.

For COVID-19 research, ‘patient’ and ‘case’ are the two largest words in Figure 7. ‘covid’ is shorthand for ‘COVID-19’– the name of the emerging infectious disease. Finally, the words ‘cases’, ‘number’ and ‘model’ are related to modelling of the disease transmission.

**Figure 8.**
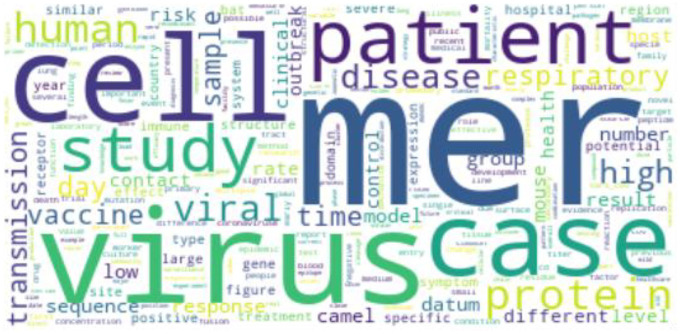
WordCloud of MERS.

For MERS research, we can see ‘mer’ and ‘virus’ in Figure 8, which refer to ‘MERS’. It indicates that a large amount of research literature mentions both MERS and coronavirus. Meanwhile, ‘patient’ and ‘case’ are related to individual-level research, while ‘camels’ indicate animal infection research. Finally, ‘transmission’ indicates research on transmission modelling.

**Figure 9.**
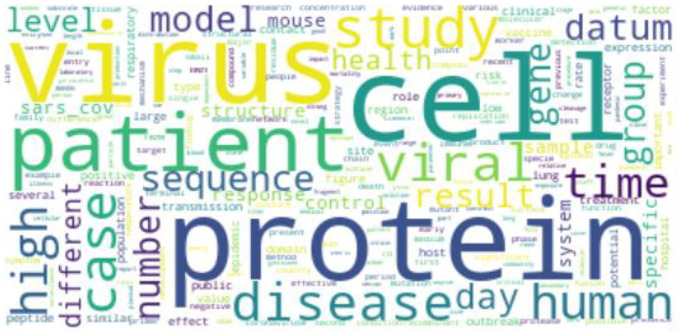
WordCloud of SARS.

For SARS research, ‘virus’, ‘protein’ and ‘cell’ are three of the largest terms in Figure 9. It indicates that many studies are related to the protein structure of SARS-CoV. ‘patient’, ‘case’ and ‘number’ are related to the infected number. ‘mouse’ is also included in the WordCloud, which is related to medical experiments using mice.

#### 4.1.3. Relevant topics for each coronavirus disease

A more interesting analysis is to identify the relevant topics for each coronavirus disease and explore the research trends for each topic. For this purpose, we use LDA to discover the research topics. Each literature could have 1 to *k* (number of topics) topics.

Table 5 shows the relevant topics for COVID-19, where each topic is presented in a row and the three dominant terms for each topic are also given. Dominant terms are those terms that could differentiate the topic from other topics. With the help of pyLDAvis, a widely used LDA visualisation tool, we selected three dominant terms from top 30 frequent terms of the topic. The column labelled ‘#’ shows the total number of studies included in each topic. ‘*β*’ is the weight of the term, which is a coefficient measuring the importance of the term in the topic. Since the original weight is very small, we give it a 10× magnification. Finally, we present the number of studies that were published throughout the analysed period (from January to April 2020). From this table, we can see that there are six research topics for the study of COVID-19. The six topics differ significantly from one another as their top three dominant terms are not the same. For example, Topic 1 is research into the infected number with 1807 literatures, while Topic 2 mainly focuses on the detection of COVID-19 with 1576 literatures. Topic 3 relates to the public health issue that related to COVID-19; it includes 1454 literatures. Topic 4 focuses on the structure or gene of the virus with 1195 literatures. 1051 literatures are categorised as Topic 5; Topic 5 is the patient care–related research. Topic 6 concentrates on the clinical symptom as well as the treatment; it contains 1705 literatures. We can conclude that all six topics are currently ‘hot’, as the frequency of published literature increases month by month (publications in May and later in 2020 are not considered as the data only include a very small part in these months).

**Table 5.** Relevant topics for COVID-19.

Topic	#	First term		Second term		Third term		January	February	March	April
		Term	*β*	Term	*β*	Term	*β*				
1	1807	case	0.36	number	0.23	model	0.19	26	199	633	949
2	1576	sample	0.16	test	0.14	reuse	0.13	14	197	547	818
3	1454	health	0.18	public	0.11	COVID-19	0.10	17	115	471	851
4	1195	cell	0.24	protein	0.22	virus	0.17	19	129	427	620
5	1051	patient	0.38	care	0.13	hospital	0.12	4	73	329	645
6	1705	patient	0.57	case	0.16	clinical	0.14	16	178	621	890

Table 6 presents the six relevant topics for the study of MERS. Topic 1 mainly concentrates on the human and animal virus and includes 869 published studies. 731 papers are focusing on the study of cellular-level research, which together comprises Topic 2. Topic 3 is the virus and vaccine–related research, containing 944 papers. Topic 4 only includes 551 publications, which is a protein structure–related topic. Topic 5 concentrates on disease detection, which includes 703 literatures. Topic 6 focuses on the infected number, which includes 1245 literatures. Table 6 also reveals the research trends for each topic. From this table, we can see that there are two bursts for all four topics: the first in 2016 and the second in 2019.

**Table 6.** Relevant topics for MERS.

Topic	#	First term		Second term		Third term		2013	2014	2015	2016	2017	2018	2019	2020
		Term	*β*	Term	*β*	Term	*β*								
1	869	virus	0.30	human	0.30	camel	0.21	33	97	101	142	134	93	139	130
2	731	cell	0.57	MERS	0.48	protein	0.34	31	69	76	121	103	97	142	92
3	944	virus	0.25	infection	0.23	vaccine	0.16	36	85	97	160	126	108	161	171
4	551	protein	0.28	structure	0.19	domain	0.14	15	36	53	78	76	67	81	145
5	703	sample	0.25	virus	0.14	positive	0.13	21	69	90	125	110	93	110	85
6	1245	MERS	0.29	case	0.25	patient	0.23	35	116	151	225	183	150	197	188

Finally, for the study of SARS, seven research topics are identified by the LDA. The results are presented in Table 7. The top three dominant terms in Topic 1 for SARS are ‘cell’, ‘infection’ and ‘mouse’, which indicates animal experiments–related research; it includes 1528 literatures. Topic 2 focuses on the public health issue related to SARS; it has 1697 literatures. Topic 3 concentrates on the structure of the virus; it includes 2128 literatures. Topic 4 pays more attention to the transmission of the disease, which includes 1881 literatures. Topic 5 contains vaccine-related research papers; it includes 2032 literatures. Topic 6 represents for the patient case–related research, which has 2179 literatures. Topic 7 focuses on the biomedical field; it includes 1184 literatures. The research trends, which could be identified by the number of published papers in a different year, are presented in Table 7.

**Table 7.** Relevant topics for SARS.

Topic	#	First term		Second term		Third term		2003–2005	2006–2008	2009–2011	2012–2014	2015–2017	2018–2020
		Term	*β*	Term	*β*	Term	*β*						
1	1528	cell	0.56	infection	0.24	mouse	0.23	392	324	190	207	174	241
2	1697	health	0.18	disease	0.13	public	0.11	507	307	220	198	204	261
3	2128	protein	0.54	cell	0.33	virus	0.13	526	480	291	273	241	317
4	1881	model	0.17	case	0.15	number	0.15	432	344	260	219	222	404
5	2032	virus	0.43	human	0.26	vaccine	0.19	463	371	233	282	291	392
6	2179	patient	0.52	case	0.20	infection	0.20	739	443	247	225	216	309
7	1184	structure	0.24	activity	0.13	site	0.13	248	237	159	178	166	196

### 4.2. Representative literature per topic

To select the most relevant studies, two metrics are considered, in the following order of priority: the number of different terms mentioned in each literature (from one to all three of the most relevant terms, displayed for each topic) and the total number of times each of the three terms occurs, regardless of the specific topic.

Table 8 shows representative publications on the six COVID-19 topics. The representative literature for Topic 1 develops a new transmission model, which integrates a global network model with a local Susceptible-Exposed-Infective-Recovered (SEIR) spreading model to predict the outbreak dynamics of the COVID-19 [51]. The study chosen to represent Topic 2 builds a deep learning model to help the screening of COVID-19 based on computed tomography (CT) images [52]. Topic 3’s representative paper offers some recommendations to the universities to help mitigate the negative effect of COVID-19 on students’ mental health [53]. Topic 4’s representative paper ‘provides a comprehensive structural genomics and interactomics road-maps of’ SARS-CoV-2 [54]. Topic 5’s representative study proposes five practical steps to prevent the spreading of infectious disease in Long-Term Resident Rooms [55]. Topic 6’s representative paper analyses ‘the clinical characteristics and laboratory findings’ of COVID-19 cases [4]. All six papers were published in 2020. Their authors are named in Table 8.

**Table 8.** Representative literature of COVID-19.

Topic	Paper	First term		Second term		Third term	
		Term	Frequency	Term	Frequency	Term	Frequency
1	Peirlinck et al. [51]	patient	12	hyposmia	10	case	7
2	Wang et al. [52]	cell	33	nasal	14	expression	13
3	Zhai and Du [53]	health	18	professional	12	patient	7
4	Cui et al. [54]	case	37	patient	29	severe	27
5	Lynch and Goring [55]	people	27	study	20	case	19
6	Zhang et al. [56]	protein	43	host	39	sequence	38

Table 9 lists the six representative studies of MERS-related research. Each study corresponds to one topic. The first representative paper investigates the factors that affect the response to infectious disease with the help of meta-analyses [57]. The second one introduces the hospital outbreak of MERS in South Korea in 2015 [58]. The third representative study discusses the potential drugs and treatments to both MERS and SARS [59]. The fourth study introduces the current understanding of MERS in 2014 [60]. Camel is doubted to be a possible source of the virus. The fifth representative paper talks about the emerging and spreading of MERS in 2012 [61]. The sixth representative paper is a review focus ‘on the origin, epidemiology and clinical manifestations of MERS-CoV’ as well as ‘the diagnosis and treatment of infected patients’ [62].

**Table 9.** Representative literature of MERS.

Topic	Paper	First term		Second term		Third term	
		Term	Frequency	Term	Frequency	Term	Frequency
1	Lee and Jung [57]	case	60	outbreak	34	mer	26
2	Park et al. [58]	time	29	sample	29	specimen	28
3	Dyall et al. [59]	mer	29	case	23	patient	20
4	Sampathkumar [60]	outbreak	19	period	17	mer	15
5	Raj et al. [61]	sequence	35	specie	34	strain	33
6	Bleibtreu et al. [62]	nanoparticle	72	cell	63	vaccine	54

Table 10 lists the seven representative studies of SARS-related research. Each study corresponds to one topic. The first representative paper focuses on the host cell of SARS-CoV [63]. The second studies the structural proteins of SARS-CoV [64]. The third study investigates the ‘clinical, radiologic, and hematologic findings of SARS patients with pneumonia’ [65]. The fourth study examines ‘whether the initial chest radiograph helps predict the clinical outcome of patients’ with SARS [66], and the answer is yes in this study. The fifth one introduces the pregnancy outcome of a woman who was exposed to the SARS [67]. The sixth one develops a new approach to help optimise the lead inhibitor of SARS-CoV [68]. The seventh representative paper conducts a comparative analysis of the transmission and epidemiological characteristics of both SARS and COVID-19 [69].

**Table 10.** Representative literature of SARS.

Topic	Article	First term		Second term		Third term	
		Term	Frequency	Term	Frequency	Term	Frequency
1	Simmons et al. [63]	cell	144	cleavage	107	site	103
2	Yuan et al. [64]	compound	15	protease	7	site	7
3	Wang et al. [65]	case	63	patient	37	animal	24
4	Chau et al. [66]	phage	69	protein	45	peptide	25
5	Rezvani and Koren [67]	virus	38	protein	20	animal	17
6	Shao et al. [68]	polymer	128	cell	114	virus	63
7	Zhang et al. [69]	infection	20	symptom	15	virus	13

### 4.3. Topic similarity analysis

**Figure 10.**
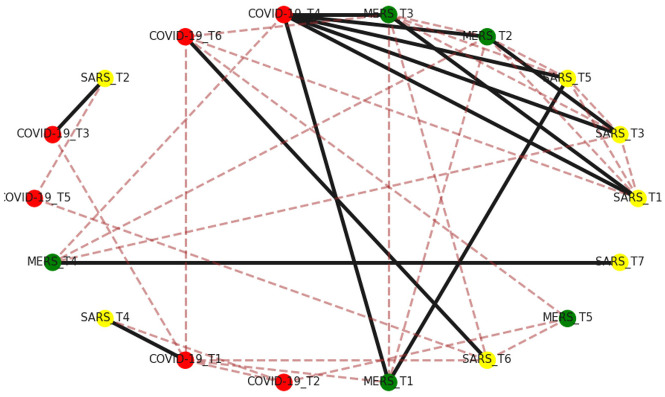
Network visualisation of topic similarity.

To examine the semantic similarity and differences among topics extracted by LDA, we use the Jaccard similarity scores to measure the similarity between pairs of topics, with a range from 0 to 1 [65]. We choose the top 30 most frequent topic terms to calculate the similarity score. In Figure 10, the red nodes are COVID-19-related topics, the green nodes are MERS-related topics and the yellow nodes are SARS-related topics (‘T’ means ‘Topic’). The edges are the similarity scores between the two topics. To simplify the structure of the network, similarity scores under 0.15 are excluded. Brown dashed lines indicate that the similarity scores are under 0.3. The black solid lines are those above 0.3. Figure 10 indicates that Topic 4 of COVID-19 is sharing much more topic terms with other topics. It is highly correlated to six topics, including Topics 1, 2 and 3 of MERS and Topics 1, 3 and 5 of SARS. What’s more, Topics 1, 2 and 3 of MERS and Topics 1, 3 and 5 of SARS are highly correlated to another two topics. They have the same number of highly correlated topics.

## 5. Conclusion and discussion

The outbreak of COVID-19, caused by SARS-COV-2, represents one of the most substantial global challenges in this century. Millions of people have been infected while hundreds of thousands have died. In response to the pandemic, a large number of academic studies and case reports have already emerged in major international scientific and medical journals. However, the huge amount of coronavirus literature makes it difficult for the health and medical community to keep up. By applying text mining and LDA to conduct a literature analysis on three coronavirus diseases – COVID-19, MERS and SARS – we illustrate that information specialists can support the health and medical community using information techniques in literature analysis. We first present the most relevant terms appearing in research on coronavirus diseases and identify the main research themes. We then uncover representative studies for each main research theme as examples to guide the health community to find appropriate literature on the target themes for these diseases. Finally, we build a novel visual concept network to show the degree of overlap, similarity and difference among these themes. This study can help the health and medical community to extract useful information and interrelationships from a mass of coronavirus literature, such as finding the structured morphology of the existing literature and uncovering research themes and representative studies. Our work also provides a methodological framework for literature analysis that can rapidly present a snapshot for any specific field of study: a very important requirement for many people, such as new entrants to a research field, researchers from other fields and policymakers, to evaluate possible opportunities for new research and development activities.

There are also some limitations to the study. First, although the data set is large, it is not possible to collect all related articles because of time and access-rights limitations. Second, we only use the most popular topic modelling method – the LDA model. Other methods, such as clustering, could supplement our research strategy. Third, we only use the full-text data and neglect the abstract data. Titles, abstracts and keywords can also provide useful information for topic modelling. We plan to conduct LDA topic modelling using abstracts in future work.

This study has many valuable implications in the future. By performing the proposed text mining framework, we can identify the most relevant search terms and generic research themes of different research topics. Besides, our study could help the health and medical community to find the appropriate studies on target themes for these diseases. What’s more, our visual concept network could visualise the similarities among the research. In the future, we plan to collect more literatures and apply more advanced techniques to support the fight against the pandemic.
